# Massive gastrointestinal haemorrhage caused by pancreatic pseudocyst complicated with Dieulafoy's disease in a child: A case report and review of the literature

**DOI:** 10.3389/fped.2022.962465

**Published:** 2022-09-08

**Authors:** Lintao Liu, Lichao Zhang, Xiaoli Zhu, Meng Li, Juan Cao, Likang Ji, Xiaoyang Qi, Weili Xu

**Affiliations:** ^1^Department of Paediatric Surgery, The Second Hospital of Hebei Medical University, Shijiazhuang, China; ^2^Department of Minimally Invasive Biliary Surgery, The Second Hospital of Hebei Medical University, Shijiazhuang, China; ^3^Department of Paediatric Surgery, People Hospital of Xingtai, Xingtai, China

**Keywords:** injury, pancreatic pseudocyst, stomach Dieulafoy's disease, massive gastrointestinal haemorrhage, child

## Abstract

**Background:**

Pancreatic pseudocyst (PPC) with massive gastrointestinal bleeding is rare, especially in children. Inadvertent intraoperative examination and damage to the gastric mucosa and malformed blood vessels by the fluid content of PPC can lead to massive bleeding, which may endanger the patient's life.

**Case presentation:**

Here, we present a case of an 8-year-old boy who was diagnosed with a massive gastrointestinal haemorrhage caused by PPC complicated with Dieulafoy's disease. At his first admission, his complaint was being hit to the stomach by the handlebar while riding bicycle 24 h before admission. After being hospitalized, he was diagnosed with pancreatic injury by abdominal CT. Conservative treatment lasted for 1 month in the Department of Pediatric Surgery. Then, a pancreatic pseudocyst was formed. Under the guidance of ultrasonic endoscopy, cyst puncture and drainage of pseudocysts through the gastric wall were performed. Unexplained hematemesis occurred 8 days after surgery. Emergency gastroscopy was performed, and abnormal submucosal vascular haemorrhage was found at the gastric fundus. Gastric Dieulafoy's disease was diagnosed. The boy underwent gastroscopic titanium clipping of abnormal arteries. He had no complications during the 3-month follow-up. Then, the patient returned to the hospital, and the stent was removed under endoscopy. No bleeding was found, and the patient was discharged. The patient recovered smoothly and was followed up for half a year without any complications, and hematological indicators were normal.

**Conclusion:**

Endoscopic ultrasonography-guided gastric puncture and internal drainage of cysts is a safe and effective surgical method for the treatment of pancreatic pseudocysts. However, at the same time, it is necessary to thoroughly and carefully explore the stomach cavity to prevent adverse consequences caused by a missed diagnosis of gastric Dieulafoy's disease or other abnormal abnormalities.

## Introduction

**Figure 1:**
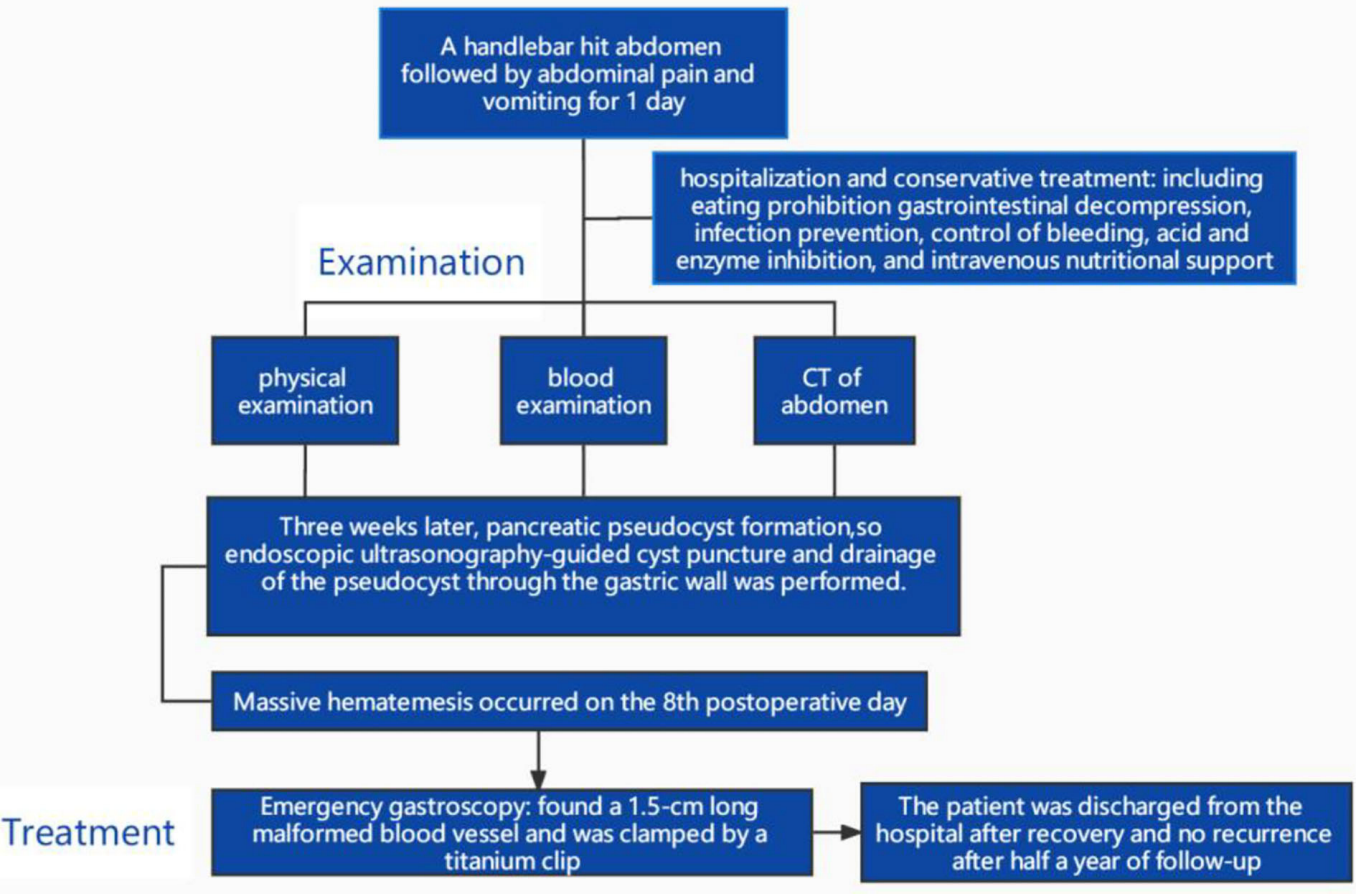
Timeline of the treatment process of the patient.

Dieulafoy's disease (DD), also known as Dieulafoy ulcer, Dieulafoy injury, or submucosal artery rupture and haemorrhage, is a rare and fatal cause of massive gastrointestinal bleeding. The disease was named after Dieulafoy, a French surgeon who reported three cases of this fatal digestive tract haemorrhage from 1896 to 1898 and described its characteristics in detail (1, 2). Since the 1990s, there have been clinical reports of this disease. In recent years, with the development and popularization of endoscopic diagnosis and treatment technology, the detection rate has increased significantly. Dieulafoy's disease can occur at any age, mostly in middle-aged and elderly, and is extremely rare in children. The clinical manifestations often depend on the lesion location. Dieulafoy's disease accounts for 1−2% of acute gastrointestinal bleeding and is present throughout the digestive tract. It is usually located in the fundus and lesser curvature of the stomach. Approximately 80−95% of lesions occur within 6 cm from the gastroesophageal junction, and they can also be seen in the esophagus (8%), duodenum (15%), empty ileum (1%), colon (2%), rectum (2%), and stomach anastomosis (1%) (2). It is also occasionally seen in the gallbladder and bronchus (3). The disease has a rapid onset without obvious warning symptoms. It often bleeds violently. Heavy bleeding can cause hematemesis and bloody stools. If left untreated, it can be life-threatening. Here, we report a rare case of massive gastrointestinal haemorrhage caused by pancreatic pseudocyst (PPC) complicated with Dieulafoy's disease in an 8-year-old boy. Figure 1 shows the timeline of the treatment process of the patient.

## Case report

**Figure 2:**
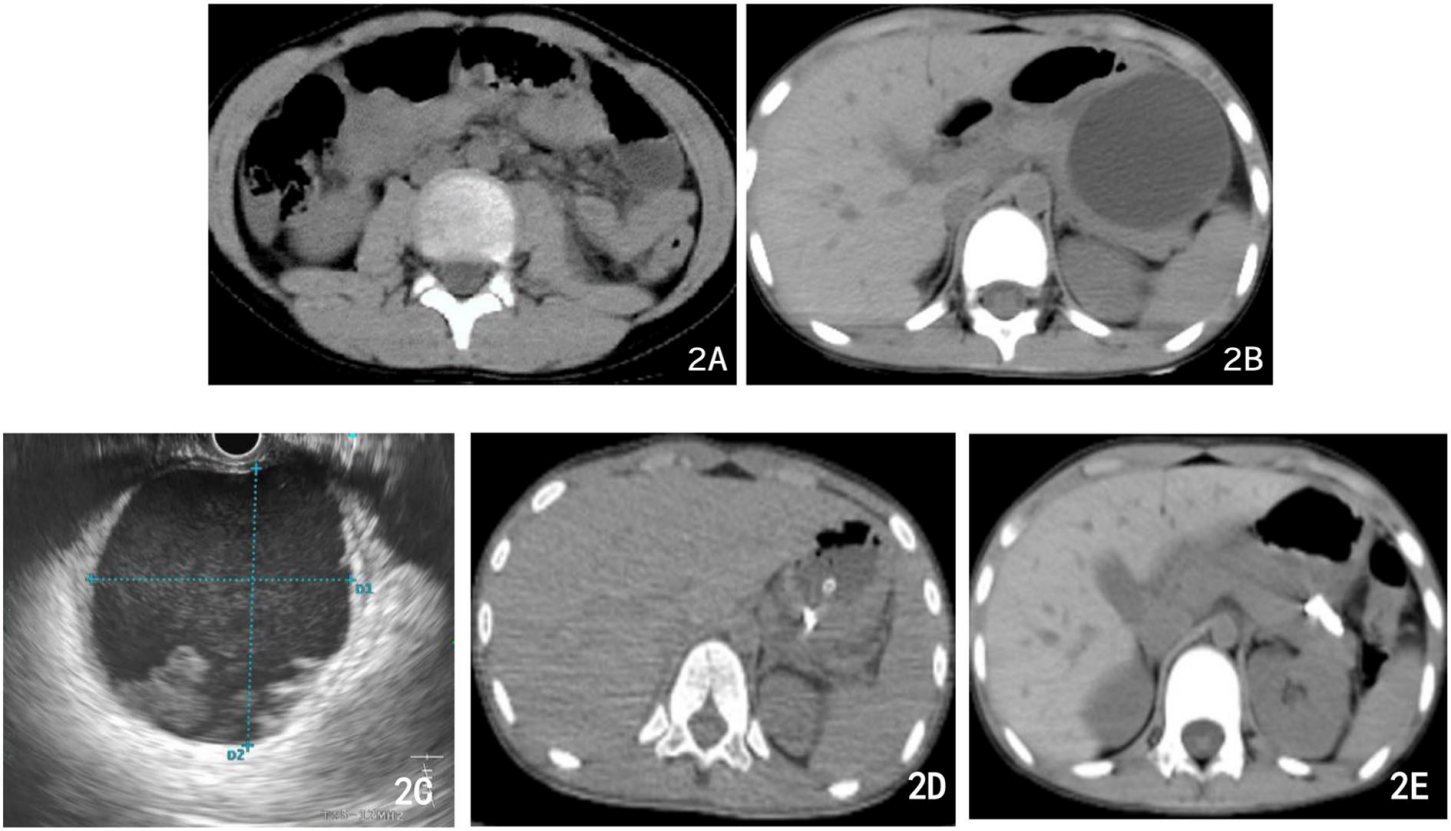
Abdominal CT imaging of different therapeutic periods. **(A)** First abdominal CT after bicycle accident: the tail of the pancreas was thickened, lumpy high-density shadows near the tail of the pancreas, and patchy isodensity shadows around (the possibility of hematocele and exudation). **(B)** Abdominal CT 3 weeks later: PPC formation with a maximum cross size of ~6.5 cm × 6.8 cm. **(C)** During the operation, the ultrasound endoscope found that the echo-free zone existed in the area, with a slightly high echo in the shape of a mass, a weak echo in the shape of a floating dot, and no blood flow signal in the area. **(D)** Postoperative abdominal CT: PPC in the tail of the pancreas was significantly reduced, with tubular shadows and little air accumulation in the corresponding area. **(E)** Abdominal CT 10 days after the previous one **(D)** multiple strip-shaped extremely high-density shadows in the tail of the pancreas and a few surrounding strips with slightly lower density.

Our patient is an 8-year-old boy. At his first admission, his complaint was being hit to the stomach by the handlebar while riding bicycle 24 h before admission. He was admitted to the Department of Pediatric Surgery after 1 day of abdominal pain with vomiting. He did not receive any special treatment before admission. Physical examination showed abdominal tenderness under the xiphoid process, mild local muscle tension, no rebound pain, a soft rest of the abdomen, and normal bowel sounds. Abdominal CT (Figure 2A) showed that the tail of the pancreas was thickened, lumpy high-density shadows could be seen near the tail of the pancreas, and patchy isodensity shadows could be seen around it, which suggested the possibility of hematocele and exudation. Blood amylase was high at a value of 206.0 U/L, and urine amylase was 1,193.0 U/L. Therefore, closed abdominal injury and pancreatic injury were diagnosed preliminarily. After admission, the patient was given conservative treatment, namely, eating prohibition gastrointestinal decompression, flucloxacillin to prevent infection, aminocaproic acid to control bleeding, famotidine and somatostatin to inhibit acid and enzyme, and intravenous nutritional support. The boy had good health, with regular vaccinations and no history of exposure to infectious diseases. There is no genetic disease in his family.

**Figure 3:**
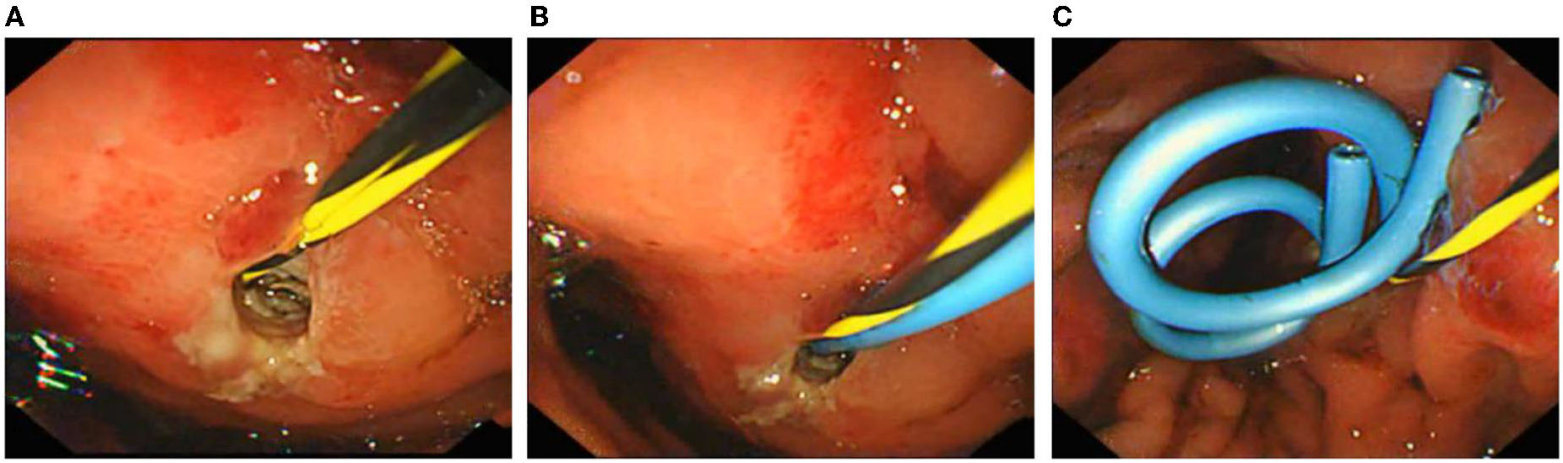
Endoscopic ultrasonography-guided cyst puncture and drainage of pseudocysts through the gastric wall. **(A)** The cyst site was punctured and dilated, and the nasal cyst tube was inserted. **(B)** Placement of the first double pigtail stent. **(C)** Two double pigtail stents and a nasal cyst tube were placed in a good position.

On the third day after admission, abdominal CT showed that there was an injury in the body and tail of the pancreas, accompanied by hematoma formation and multiple exudative changes around the pancreas, and the hematoma had grown. Then, conservative treatment was continued for 3 weeks. Finally, abdominal CT was reviewed and suggested PPC formation with a maximum cross size of ~6.5 cm × 6.8 cm, accompanied by a small amount of peripheral exudation and effusion (Figure 2B). The patient developed significant abdominal pain, nausea, and vomiting because of the compression of the cyst on the stomach wall. We considered that the PPC was too large to absorb and shrink by itself, so endoscopic ultrasonography-guided cyst puncture and drainage of the pseudocyst through the gastric wall was performed. The boy and his parents agreed and supported our treatment plan. During the operation, endoscopic ultrasound entered the stomach and found that the gastric wall was edematous, and the mucosa at the posterior wall of the gastric body was compressed. Ultrasound showed that there was a cystic area in the body and tail of the pancreas, with a size of 5.8 cm × 6.2 cm. The echo-free zone existed in the area, with a slightly high echo in the shape of a mass, a weak echo in the shape of a floating dot, and no blood flow signal in the area (Figure 2C). Under the guidance of ultrasound Doppler, the blood vessels were avoided, a 19 G puncture needle was punched on the cystic area, and the brown liquid was pumped back. The guide wire was indwelled inside the needle into the cyst cavity, and the needle was withdrawn. A three-cavity sphincter incision was performed along the guide wire, and a 10 Fr dilated probe was used to expand the puncture site. A large amount of brown liquid was seen flowing out, and a second guide wire was inserted along the dilated probe. The probe rod was withdrawn, and the puncture channel between the stomach and the cyst was expanded to 15 mm by using a progressively dilated water sac along the guide wire. The dilated water sac was withdrawn, and two double pigtail stents with a diameter of 7 Fr and a length of 5 cm were placed in the cyst cavity along the guide wire. The other end was placed in the stomach cavity. A nasal cyst tube with a diameter of 7 F was placed in the cyst along the other guide wire, and the catheter was extracted from the nasal cavity (Figures 3A–C). Postoperative recovery was smooth, and no special discomfort was reported.

**Figure 4:**
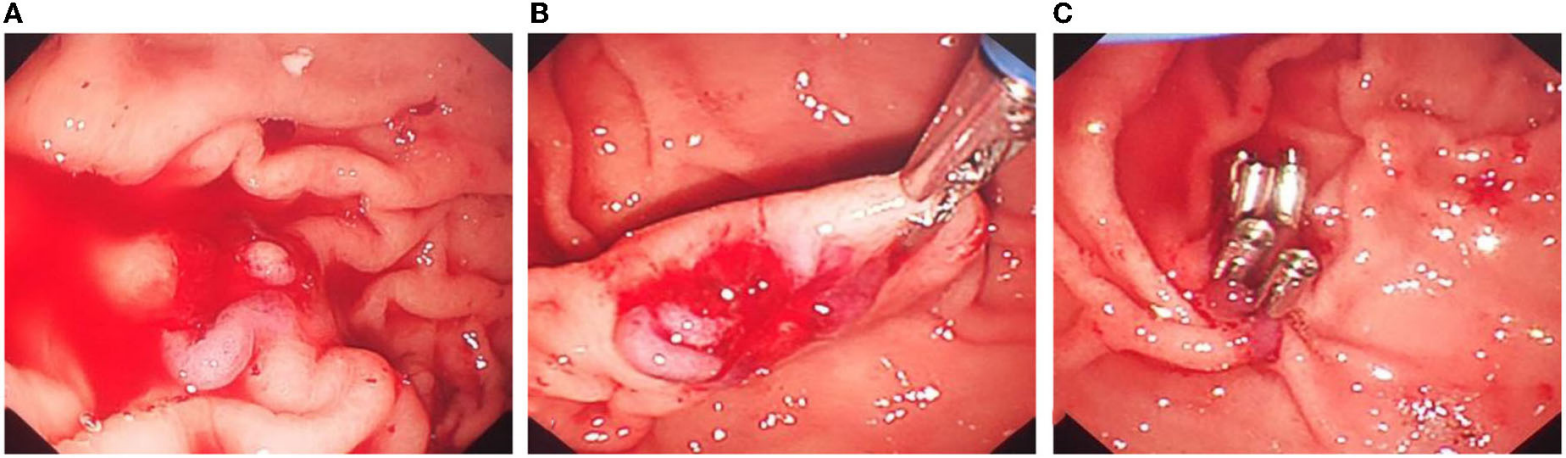
Titanium clip closure of abnormal blood vessels in the Dieulafoy's disease under gastroscopy. **(A)** After cleaning the old bleeding in the gastric cavity, continuous bleeding of exposed malformed blood vessels can be seen. **(B)** There was no obvious abnormality in the mucosa around the lesion, and the exposed artery was bleeding quickly. **(C)** Titanium clips were used to clip the exposed abnormal blood vessels to stop bleeding.

However, on the 8th day after surgery, the child vomited blood, and the blood clot was bright red with a volume of ~220 ml. Then, he vomited blood twice again, ~220 ml and 180 ml, respectively. Therefore, severe acute upper gastrointestinal haemorrhage was considered, and an emergency gastroscopy was performed. Gastroscopy showed a large amount of bloody gastric juice and old blood clots in the gastric cavity. No obvious active bleeding was found at the puncture and anastomosis, and the stent and drainage tubes were in a good position. After the blood clot was cleaned up by foreign body forceps, a 1.5-cm long malformed blood vessel was observed in the submucosa of the greater gastric curvature, with a blue surface and much blood oozing. The blood vessel was clamped by a titanium clip. Then, endoscopy detection was continued to the duodenum, and no obvious bleeding or oozing was found. As a result, the intraoperative supplementary diagnosis was gastric Dieulafoy's disease (Figures 4A–C). Postoperative abdominal CT (Figure 2D) showed that cystic lesions in the tail of the pancreas were significantly reduced, with tubular shadows and little air accumulation in the corresponding area, and a small amount of exudate could be seen. Abdominal CT was re-examined 10 days later (Figure 2E). There were multiple strip-shaped extremely high-density shadows in the tail of the pancreas and a few surrounding strips with slightly lower density, and the surrounding exudate was less than before.

**Figure 5:**
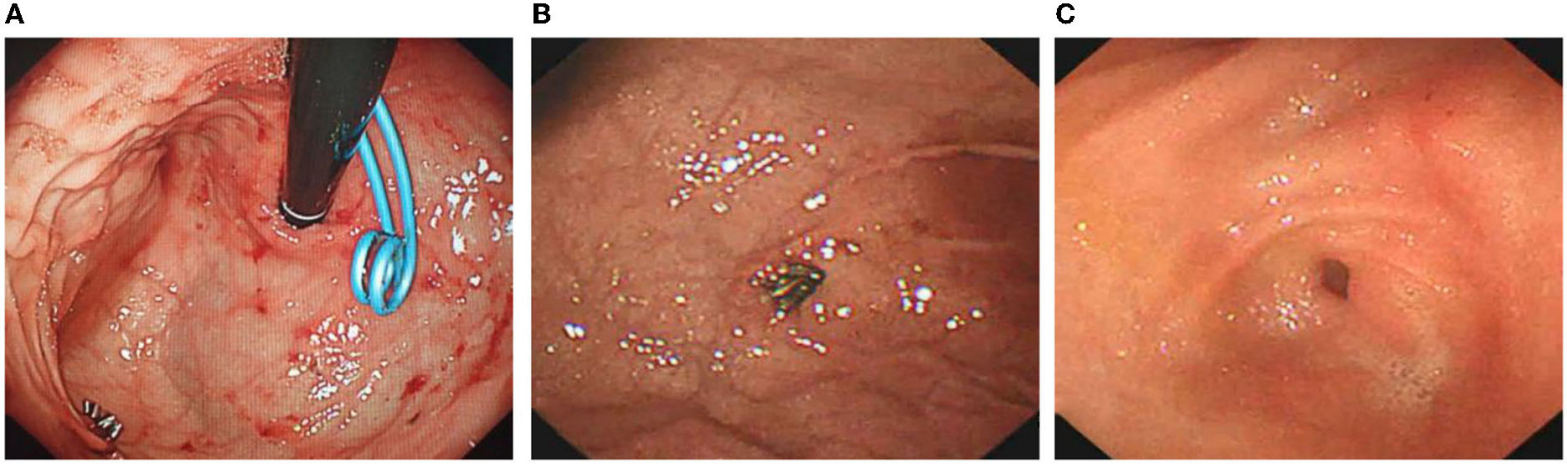
Removal of stents under gastroscopy and review of Dieulafoy's disease. **(A)** Gastric mucosa was smooth. **(B)** Titanium clips on the greater curvature side of the gastric fundus were well fixed, and no bleeding or other lesions were observed. **(C)** The double pigtail stents were removed, and no abnormalities were found.

The patient was discharged half a month after surgery. In total, 3 months later, the patient returned to the hospital for transendoscopic stent removal (Figures 5A–C). Under endoscopy, the gastric mucosa was smooth, and peristalsis was good. The titanium clip was well fixed in the greater gastric curvature without bleeding or other lesions. Two pairs of pigtail stents were extracted from the posterior wall of the stomach. The stent was removed with a snare device, and the operation was successful without bleeding or oozing at the puncture site. After half a year of follow-up, the child recovered smoothly.

## Discussion

Pancreatic pseudocyst (PPC) is mainly caused by acute and chronic inflammation of the pancreas, obstruction or injury of the pancreatic duct, and other abnormal fluid retention in or around the pancreas, which is wrapped by fibrous tissue to form capsular structures. PPC in children are relatively rare and are mostly caused by pancreatic trauma, accounting for 60−70% of all the cases (4). PPC in children generally occurs between 2 weeks and 14 months after pancreatic injury (4). The treatment of children with PPC includes conservative treatment, surgery, and endoscopic interventional therapy. At present, there is no unified conclusion on the choice of the best treatment for PPC. Clinicians need to make a comprehensive judgment based on the clinical characteristics of the children so as to select an appropriate individualized treatment plan (4–9). It has been pointed out in the literature that early clarification of the classification of pancreatic injury and whether the pancreatic duct and cyst are connected plays a crucial role in the selection of treatment methods. For PCC caused by trauma, external drainage is preferred; for PCC caused by pancreatitis, internal drainage should be advocated (10, 11). Endoscopic ultrasound-guided pseudocyst puncture and drainage have gradually become the first-line treatment for PPC due to its advantages of less trauma, fewer complications, short hospital stay, and low cost (12). This procedure needs to be performed when the cyst wall is mature and tough to reduce the risk of surgical failure. However, there are still many problems, such as the timing of puncture and drainage and stent selection, whether to place a nasal drainage tube, and stent removal time. More clinical studies are needed to further optimize, improve, and standardize these principles (8, 10, 13, 14).

In this case, the operation was performed at the stable stage of the cyst, and the postoperative recovery was smooth. Abdominal pain, vomiting, and other symptoms were significantly relieved, blood and urine amylase and lipase decreased significantly compared with the preoperative levels, and ultrasound showed only a small amount of fluid around the pancreatic tail. However, on the 8th day after the operation, the child suddenly developed a large amount of hematemesis, so an emergency gastroscopy was performed. During the operation, the stomach Dieulafoy's disease was diagnosed because an unexpected haemorrhage was found in the submucosal malformed blood vessels of the stomach, and haemorrhage was relieved by a titanium clamp.

Dieulafoy's disease occurs mostly in male adults and is rare in children. It is mostly sporadic and characterized by sudden, interstitial, and repeated bleeding (1, 3, 15, 16). The incidence of Dieulafoy's disease in children has not been determined, and its causes are still unknown (3, 16). Although rare, it may also occur in newborns, which supports the innate origin of the disease (17). Studies have shown that irritating food, alcohol, nonsteroidal anti-inflammatory drugs, stress, hypertension in elderly patients, and decreased vascular compliance caused by atherosclerosis can all induce Dieulafoy's disease (1–3).

Dieulafoy's disease occurs mostly in the gastrointestinal tract, which is relatively obscure and complex and is an easily missed diagnosis (16). A case of colon Dieulafoy's disease was diagnosed during the third colonoscopy because of multiple intestinal bleeding, and retrospective observation found that the first two examinations were missed (2). A 56-year-old man developed a rare duodenal Dieulafoy's disease after a Roux-en-Y gastric bypass operation (18). Similarly, a 69-year-old man underwent antrectomy and Roux-en-Y reconstruction for duodenal neuroendocrine tumors, which resulted in immediate postoperative bleeding and was diagnosed as anastomotic Dieulafoy's disease by endoscopic examination (19). Dieulafoy's disease after Roux-EN-Y surgery is not accidental. The patient may have congenital vascular malformation, which is the basis of Dieulafoy's disease. The gastrointestinal tract is reconstructed by Roux-en-Y surgery. Although measures such as preventing reflux valve are taken during the operation, gastroduodenal reflux is often present after the operation. Exposure of the stomach or duodenum to unaccustomed acid/base levels, combined with the trauma of the operation itself, postoperative stress, and gastrointestinal damage caused by prolonged fasting, may be predisposed factors for Dieulafoy's disease, as demonstrated in the case reported here.

In addition, cases of Dieulafoy's disease accompanied by canceration have been reported (20), but whether the two are necessarily related remains to be discussed. Dieulafoy's disease occurs frequently but is not limited to the gastrointestinal tract. There are also many reports of bronchial Dieulafoy's disease in children, which is characterized by arterial rupture and haemorrhage caused by malformation of bronchial submucosa (21–25). The diagnosis and treatment of bronchial Dieulafoy's disease have provided us with many experiences and lessons for the diagnosis and treatment of the digestive tract Dieulafoy's disease. By analyzing the previous (after 2010) and present cases, the clinical characteristics are listed in Table 1.

**Table 1:** Literature review on Dieulafoy's disease in children.

**Case**	**Age/sex**	**Presentation**	**Site**	**Diagnosis**	**Treatment**	**Recurrence**
1 (15)	18 months/F	Haematemesis and melena with progressively worsening anemia	Stomach	Oesophagogastroduodenoscopy	Apply two elastic bandages	No
2 (15)	8 years/M	Haematemesis and melena.	Stomach	Oesophagogastroduodenoscop	Treatment with three endoscopic haemoclips	No
3 (16)	6 years/F	Haematochezia	sigmoid colon	Colonoscopy	Cauterize	No
4 (17)	Neonate/M	Haematemesis and melena	Stomach.	GI endoscopy	Epinephrine injection	No
5 (21)	9 months/M	Intermittent haematemesis	Right lower lobe	Bronchoscopy	Bronchial artery embolism (BAE)	No
6 (23)	11 year/M	Chest discomfort and massive haemoptysis	Right lower lobe	Bronchial artery radiographic	Bronchial artery embolism (BAE)	No
7 (24)	7 years/F	Massive haemoptysis	Right bronchus intermedius. (RBI)	Computer tomography angiography (CTA), a repeat bronchoscopy and angiography of the bronchial artery	A sleeve resection of the RBI was performed over angiographic embolization	No
8 (25)	8 years/M	Severe haematemesis	Right lower lobe	Fibreoptic bronchoscopy	Bronchial artery embolisation (BAE)	No
9 (26)	5 years/M	Melaena and hypovolaemic shock	Stomach	GI endoscopy	Epinephrine injection and the application of three haemostatic clips	No
10 (27)	2 years/F	Haematemesis,	Stomach	GI endoscopy	High frequency electrocoagulation	No
11 (28)	13 months/F	Melena	The proximal part of the postbulbar region of the duodenum	Oesophagogastroduodenoscopy	1 ml of 5% glucose solution injection, clipping, and coagulation by installed clips	Yes, the first day after operation, Migration of endoscopic clips was identified through the abdominal X-ray, then endovascular embolization was performed
12 (29)	9 years/F	—	Jejunum	Postoperative pathology report	—	No
13 (30)	13 years/M	Massive haemoptysis	Right lower lobe	Bronchoscopy and Bronchial artery angiography	Bronchial artery embolisation (BAE)	Yes, 3 months later, Thoracotomy with bilobectomy(the right middle and lower lobe)
14 (31)	13 years/M	Intermittent haemoptysis	Right lateral basal bronchus and the subcarina of the right lateral and posterior basal bronchi	Flexible bronchoscopy	Bronchial artery embolisation (BAE)	Yes, thoracotomy with surgical resection of the right middle and lower lobes
15 (32)	8 months/M	Haemoptysis	Right upper lobe	Postoperative pathological diagnosis	Right upper lobe lobectomy after two failed bronchial artery embolization	Yes, 52 months later, haemoptysis again, conservative treatment is effective
16 (33)	2 months/M	Haematemesis,	Stomach	Gastroscopy	Place a haemostat	No
17 (34)	6 years/M	Haematemesis, melena and Haemorrhagic shock	Stomach	GI endoscopy	Clamped with haemoclips	No
18 (35)	Neonate/M	Fresh blood was aspirated from the stomach	Stomach	Endoscopy	Apply haemoclip	Died of respiratory complications 51 days after birth
19 (36)	14 years/M	Fatigue, nausea and syncope	The second portion of the duodenum	Oesophagogastroduodenoscopy (EGD)	Mbolisation of the bleeding branch with 50% N-butyl cyanoacrylate	No
Current	8 years/M	Haematemesis	Stomach	GI endoscopy	Clamped by titanium clip	No

There are many diagnostic methods for Dieulafoy's disease, including endoscopy, angiography, isotope scan, enhanced CT (especially CTA), etc. (2, 3), and endoscopy has become a recognized first-line diagnosis and treatment method (3, 17, 26, 37, 38). There are three types of endoscopic treatment: (1) injection therapy, including local injection of epinephrine and hardener; (2) coagulation therapy, namely, thermal coagulation, electrocoagulation, argon ion coagulation, etc.; and (3) Mechanical treatment, including endoscopic hemostatic clamp and ligation. Almost all the endoscopic hemostasis methods have been reported to successfully control bleeding (2, 17, 27, 39, 40). Angiography has also been recommended by some scholars as a first-line diagnosis and treatment method, which can not only confirm the diagnosis but also perform emergency arterial embolization and drug infusion hemostasis treatment (2, 28, 41). Studies have indicated that the combination of two or more treatments can significantly improve the success rate of surgery and reduce the risk of rebleeding (42, 43). Emergency surgical treatment should be considered when the efficacy of the above treatment methods is not good, and the patient has life-threatening bleeding (44). Therefore, there is no unified consensus on the treatment of Dieulafoy's disease. In actual clinical work, many factors, such as medical conditions, doctors' professional ability, patients' willingness, and condition evaluation, should be fully considered to make the most beneficial choice for patients.

This case had a clear history of trauma, and the location, symptoms, signs, and CT examination all suggested pancreatic injury. After conservative treatment, PPC was formed. Therefore, before the operation, endoscopic ultrasonography focused on the detection of PPC and their adjacent gastric walls but ignored the possibility of “Dieulafoy ulcer” on the far side of the gastric fundus and did not conduct a comprehensive and careful exploration of the gastric wall, which is also the lesson leading to the first postoperative gastrointestinal bleeding in this case. During the second operation, we learned the lesson and carried out a comprehensive and careful scan of the gastric wall, and no other lesions were found.

In this pediatric patient, Dieulafoy's disease occurs after the PPC operation which is a rare situation. Analyzing the reasons, it may be that malformed vessels exist in the submucosa of the greater curvature of the stomach. Although the PPC and malformed vascular lesions were far apart, the fluid content of the PPC might have damaged both the gastric mucosa on the vessel and the malformed blood vessel. When pancreatic fluid, blood, and necrotic tissue flowed into the gastric cavity, causing active vascular bleeding. Therefore, this gives clinicians a very important warning; that is, surgical trauma and corrosion of digestive fluids can induce the occurrence of Dieulafoy's disease when coelomic operations, especially for PPC and other gastrointestinal surgeries, are performed. The key to preventing it is the comprehensive and careful examination of the digestive tract and the elimination of abnormal blood vessels in the body cavity to avoid adverse consequences.

## Data availability statement

The original contributions presented in the study are included in the article/supplementary material, further inquiries can be directed to the corresponding author/s.

## Ethics statement

The studies involving human participants were reviewed and approved by Ethics Committee of the Second Hospital of Hebei Medical University and affiliated to the Second Hospital of Hebei Medical University. The patients/participants provided their written informed consent to participate in this study. Written informed consent was obtained from the individual(s), and minor(s)' legal guardian/next of kin, for the publication of any potentially identifiable images or data included in this article.

## Author contributions

LL wrote the manuscript and assisted in the operation. LZ and WX completed the surgery and reviewed and agreed to submit the paper. XZ and ML collected the patient data and followed up. JC, LJ, and XQ worked on editing the pictures. All authors contributed to the article and approved the submitted version.

## Conflict of interest

The authors declare that the research was conducted in the absence of any commercial or financial relationships that could be construed as a potential conflict of interest.

## Publisher's note

All claims expressed in this article are solely those of the authors and do not necessarily represent those of their affiliated organizations, or those of the publisher, the editors and the reviewers. Any product that may be evaluated in this article, or claim that may be made by its manufacturer, is not guaranteed or endorsed by the publisher.
